# Novel metabolomic profile of subjects with non-classic apparent mineralocorticoid excess

**DOI:** 10.1038/s41598-021-96628-6

**Published:** 2021-08-25

**Authors:** Alejandra Tapia-Castillo, Cristian A. Carvajal, Xaviera López-Cortés, Andrea Vecchiola, Carlos E. Fardella

**Affiliations:** 1 Department of Endocrinology, School of Medicine, Pontificia Universidad Católica de Chile, Diagonal Paraguay 362, Piso 4, 8330077 Santiago, Chile; 2 Millennium Institute on Immunology and Immunotherapy (IMII-ICM), Santiago, Chile; 3 Centro Traslacional de Endocrinología UC (CETREN-UC), Santiago, Chile; 4 Department of Computer Science and Industries, Faculty of Engineering Science, Universidad Católica del Maule, Talca, Chile

**Keywords:** Adrenal gland diseases, Translational research

## Abstract

Nonclassic apparent mineralocorticoid excess (NC-AME) is proposed as a novel clinical condition with a mild phenotypic spectrum that ranges from normotension to severe hypertension. This condition is mainly characterized by a high serum cortisol to cortisone ratio (F/E) and concomitant low cortisone (E), however further metabolic changes in NC-AME have not been studied. A cross-sectional study was performed in a primary-care cohort of 396 Chilean subjects, which were classified in two groups: NC-AME (n = 28) and healthy controls (n = 27). A discovery study based in untargeted metabolomics assay in serum samples from both groups was performed by UPLC-Q-TOF/MS. Global metabolomic variations were assayed by principal component analysis and further compared by orthogonal partial least-squares discriminant analysis (OPLS-DA). NC-AME subjects exhibited higher values of blood pressure, fractional excretion of potassium, and lower plasma renin activity and urinary sodium to potassium ratio. Metabolomic analyses showed 36 differentially regulated metabolites between NC-AME and control subjects. A ROC curve analyses identified eight metabolites with high discriminatory capacity between NC-AME and control subjects. Moreover, gamma-l-glutamyl-l-methionine sulfoxide and 5-sulfoxymethylfurfural, exhibited significant association with cortisone, which are potential biomarkers of NC-AME, however further assays should elucidate its biological role in setup and progression of this phenotype.

## Introduction

Apparent mineralocorticoid excess (AME) syndrome is an infrequently occurring autosomal recessive disorder caused by mutations in coding regions of the HSD11B2 gene^1^, and AME is characterized by the activation of the mineralocorticoid receptor (MR) by cortisol. It is known that cortisol can bind to MR with equal affinity to aldosterone, leading to the same effects as primary aldosteronism (PA)^2,3^. However, under normal conditions, activation of the MR by cortisol does not occur because the 11β-hydroxysteroid dehydrogenase type 2 (11β-HSD2) enzyme inactivates cortisol in cortisone and thus prevents cortisol from binding to the MR^4^. In the literature, there is clear evidence demonstrating that a total deficiency of the 11β-HSD2 enzyme triggers severe arterial hypertension that is characterized by a childhood onset and is associated with suppressed plasma renin activity (PRA), low aldosterone levels and hypokalemic alkalosis^5–8^.

Recent studies by our group identified mild forms of AME syndrome that we named nonclassic apparent mineralocorticoid excess (NC-AME), which is proposed to be a condition with a wider and milder phenotypical spectrum than was identified for AME^5,7,9–12^ and is primarily characterized by a high F/E ratio and low cortisone^9^. In addition, NC-AME patients have higher levels of inflammatory markers, microalbuminuria and plasminogen activator inhibitor-1 (PAI-1), and they have high sensitivity c-reactive protein (hs-CRP). To date, the cause of NC-AME has not been elucidated. However, we recently determined that NC-AME could be associated with minor genetic defects (i.e., heterozygous pathogenic variant or polymorphisms)^9^ or epigenetic modifications such as miRNAs^13^. Moreover, salt intake, glycyrrhetinic acid-like factors (GALFs) and environmental factors^5^ have been hypothesized to be factors that deregulate the expression and activity of the HSD11B2 gene.

Previous studies described the presence of inhibitor substances of the 11β-HSD2 enzyme in human urine that include glycyrrhetinic acid (GA) like-factors (GALFs), such as the licorice derivative, which affect the adequate metabolism of cortisol^14^. A large proportion of patients with essential hypertension likely exhibit endogenous GALF-like inhibitors^14^, particularly endogenous allo-3α-5α-reduced pathway steroidal products, corticosterone and 11-dehydrocorticosterone^15^, derivatives of progesterone and adrenocorticosteroids^16,17^, 11β-OH-testosterone and 11-keto-testosterone, which are potent inhibitors of 11β-HSD2 dehydrogenase activity. Moreover, several publications have showed the effects of inhibition of 11β-HSD2 by others environmental inhibitors, including licorice, GA, carbenoxolone and others previously described^17–20^.

Metabolomics can be employed to detect global metabolite profiles^21^, which represent the endpoint of all metabolic activities and help to characterize various biological and physiological processes. Previous studies of untargeted metabolomics in resistant hypertension have shown changes in metabolite levels related to fatty acid, lipid, amino acid and purine metabolism^22^, showing that metabolomics helps to elucidate the metabolites that may influence the physiopathology associated with drug-resistant hypertension.

In the present study, we performed an untargeted metabolomic approach to elucidate novel metabolite profiles in subjects with NC-AME and also get insights into the biological mechanisms underlying NC-AME condition, which support prospective identification of novel biomarkers associated with this phenotype.

## Subjects and methods

### Subjects

This study was designed as a cross-sectional study in a Chilean adult cohort of 396 subjects between 18–60 years of both genders, with a similar socioeconomic status and ethnicity; these subjects had been previously recruited from primary care centers in Santiago, Chile. The study was performed according to the principles of the Declaration of Helsinki. A written informed consent was signed by all participants. Consent and ethics approval for the recruitment of patients and samples is included in the certificate of approval CEC-MEDUC 14-268 and 12-207 of the Faculty of Medicine, Pontificia Universidad Católica de Chile.

The subjects enrolled in this study meet the following criteria: absence of a history of chronic pathologies, such as renal failure, heart failure, diabetes mellitus, chronic liver damage, and endocrinopathies, and drugs affecting the PRA and aldosterone to PRA ratio (ARR)^23,24^. Subjects who received glucocorticoids (e.g., cortisol and prednisone) or mineralocorticoids (e.g., fludrocortisone) less than 2 months before the start of the study were also excluded, since both affect the aldosterone, PRA or urinary free cortisol (F) levels.

### Clinical characteristics and biochemical assay

All subjects in this study had a clinical record and physical examination that included age, height, weight, body mass index (BMI) and blood pressure (BP)^13^. BP measurements were obtained from the right arm at consecutive 5-min intervals using an oscillometric method (Dinamap CARESCAPE V100, GE Healthcare, Medical Systems Information Technologies, Milwaukee, WI), with the subjects remaining in a seated position. Hypertension-AHA guidelines were followed to identify blood pressure categories in our cohort^25^.

After overnight fasting, basal blood samples were obtained between 08:00 and 10:00 AM. A biochemical profile was performed, including measurements of urine and plasma creatinine, electrolytes (sodium and potassium), aldosterone and PRA. Serum aldosterone and PRA were measured by radioimmunoassay using a commercial kit (Coat-A-Count Kit; Siemens, Los Angeles, CA and DiaSorin, Stillwater, MN, respectively). Serum cortisol and cortisone were quantified using liquid chromatography associated with tandem mass spectrometry (LC–MS/MS) and were validated according to the parameters suggested by the FDA and the CLSI using deuterated internal F and E standards (cortisol-d4 and cortisone-d2) in an Agilent 1200 Series HPLC unit coupled to an ABSciex 4500 QTrap mass spectrometer. All plasma, serum and urine (spot and 24-h collection) samples were used immediately or were stored at − 80 °C.

After the exclusion criteria were applied, we studied 28 NC-AME subjects (7.1%), who were identified according to previously described parameters^9^. Briefly, NC-AME subjects have a low level of serum cortisone (E) (E ≤ 2.1 μg/dl) and a high cortisol to cortisone (F/E) ratio (F/E ≥ 4.4)^9^. The NC-AME subjects were compared with a control group of 27 normotensive subjects with BMI values less than 35 kg/m^2^, no clinical risk factors and no evident diagnosis of genetic diseases or secondary diseases. Statistical analyses were performed with both groups of subjects similar in age, gender and BMI.

### Methods

#### Sample preparation for metabolomic assays by LC–MS

A volume of 100 µL of serum sample was thawed and extracted with 300 μL of methanol and 5 μL of DL-o-chlorophenylalanine (2.8 mg/mL) (internal standard (IS)) with 30 s of vortexing. Then, all samples were kept at − 40 °C for 1 h. After that samples were vortexed for 30 s and centrifuged at 12,000 rpm and 4 °C for 15 min. Finally, 200 μL of supernatant was transferred to a vial for LC–MS analysis. Quality control (QC) samples were used to evaluate the methodology. The same amount of extract was obtained from each sample and mixed as QC samples. The QC samples were prepared using the same sample preparation procedure^26^.

#### Untargeted metabolomics by UPLC–TOF–MS

Analyte separation was performed by liquid chromatography in an Ultimate 3000LC combined with Q Exactive MS (Thermo) and screened with a ESI–MS (targeted MS/MS mode). The LC system is composed of a Thermo Hyper gold C18 (100 × 2.1 mm 1.9 μm) with an Ultimate 3000LC. The mobile phase is composed of solvent A (0.1% formic acid–5% acetonitrile–water) and solvent B (0.1% formic acid–acetonitrile) with a gradient elution (0–1.5 min, 100–80% A; 1.5–9.5 min, 80–0% A; 9.5–14.5 min, 0% A; 14.5–14.6 min, 0–100% A; and 14.6–18 min, 100% A)^27^. The flow rate of the mobile phase was 0.3 mL min^−1^. The column temperature was 40 °C, and the sample manager temperature was set at 4 °C^26^. The mass spectrometry parameters in ESI+ and ESI− mode are listed as follows: ESI+: Heater Temp 300 °C; Sheath Gas Flow rate, 45 arb; Aux Gas Flow Rate, 15 arb; Sweep Gas Flow Rate, 1 arb; spray voltage, 3.0 kV; Capillary Temp, 350 °C; S-Lens RF Level, 30%. ESI-: Heater Temp 300 °C, Sheath Gas Flow rate, 45 arb; Aux Gas Flow Rate, 15 arb; Sweep Gas Flow Rate, 1 arb; spray voltage, 3.2 kV; Capillary Temp, 350 °C; and S-Lens RF Level, 60%^26,27^.

At the beginning of the sequence, we run four QC samples to avoid small changes in both chromatographic retention time and signal intensity. The QC samples are also injected at regular intervals (every ten samples) throughout the analytical run.

#### Metabolomic data analysis

The raw data were acquired and aligned using Compound Discover software version 3.0 (Thermo Q Exactive Platform), which use mzLogic scoring algorithm that combines mzCloud™ similarity searching (MS2 and MSn) with structure similarity matching to rank putative database results. The identification of Compound Discovery is based on retention time (RT), MS1 and MS2. Ions from both electrospray ionization negative (ESI−) and positive (ESI+) were merged and imported into the SIMCA-P program (version 14.1) to enable multivariate analysis. The data was normalized by the area sum of all metabolites in each sample. The peak area percentage of each metabolite was calculated and the area percentage of each metabolite multiply by 1 million. After alignment and normalization, the relative standard deviation (RSD%) was applied to evaluate the reproducibility of the metabolomics analysis by formula ((SD*100)/Mean) (Supplemental Fig. 1A,B,C).

#### Clustering analyses of metabolites

Principal component analysis (PCA) is first used as an unsupervised method, providing an overview of the data. The outlier observed were not handled since were not considered influential points. Supervised regression modeling is then performed on the data set by use of partial least squares discriminant analysis (PLS-DA) or orthogonal partial least squares discriminant analysis (OPLS-DA). The OPLS-DA allow us to identify, which variables have the largest discriminatory power, i.e. which variables contribute to the model. Then, the metabolites are filtered and confirmed by combining the results of the variable importance in projection (VIP) values (VIP > 1.5) and a significant unpaired t-tests (*p* < 0.05)^27^. False Discovery rate (FDR) based on Benjamini Hochberg method was used for adjusting *p* values toward multiple hypothesis testing (FDR < 0.05).

The PLS-DA and OPLS-DA model were validated by performing permutation tests (n = 200) (Supplementary Table 1). The quality of the fitting model can be explained by R2 and Q2 values. R2 displays the variance in the model and indicates the quality of the fit. Q2 displays the variance in the data, indicating the model’s predictability^28^ (Supplementary Table 1).

#### Pathway analysis of metabolites

Pathway analysis was performed based on the KEGG and MBRole databases. All significant metabolites were imported to obtain categorical annotations, including pathways, enzyme interactions and other biological annotations.

#### Discriminatory analyses of NC-AME condition by ROC curve

A logistic regression (LR) with bootstrapping was performed with all significant metabolites (n = 36) in order to have a predictive model suitable to discriminate the NC-AME from controls. The most predictive and significant (*p* < 0.05) metabolites to identify this phenotype, beside the receiver operating characteristic (ROC) curve analyses (AUC > 0.65 and *p* < 0.0001) were used to build the model. To explore the predictive value of these metabolites, a supervised algorithm of LR with nested cross-validation (CV) were used to train and validate the model. A nested CV is a technique that creates multiple train-test splits. It was used to obtain robust estimates of model predictive performance and avoid overfitting. We used ten fold stratified nested CV, where at each iteration 9 of the folds were used in the inner loop to train the algorithm, and the 10th fold was used in the outer loop to test the trained model (Supplemental Fig. 2). Implementations were done by using the scikit-learn library version 23.0.0^29^ and Python language programming. Model’s performance was evaluated according to the metrics of accuracy, sensitivity, specificity and area under the ROC curve. The 95% confidence intervals were estimated for all metrics.

To define the clinical significance of the metabolites in the NC-AME condition, the variables included in the final model was restricted to the significant metabolites and the most relevant clinical parameters (e.g., cortisone and cortisol to cortisone ratio)^9^. The association between the best clinical predictor (e.g., serum cortisone) and metabolites was also assessed using a multivariate linear regression analysis. The analyses were performed with SPSS 20 and GraphPad Prism v9.0 software.

## Results

### Clinical and biochemical profile of NC-AME subjects

The baseline characteristics of both 28 NC-AME subjects and 27 healthy controls are shown in Table 1. NC-AME subjects had significantly higher values of SBP (*p* < 0.0001), DBP (*p* < 0.0001), fractional excretion of potassium (FEK) (*p* = 0.03) and serum F/E (*p* = 0.01) and a lower PRA (*p* = 0.005), serum cortisone (*p* < 0.0001) and urinary Na/K ratio (*p* = 0.004). Both groups exhibited comparable serum aldosterone and cortisol levels (Table 1).

**Table 1:** Clinical and biochemical characteristics of the studied subjects.

	NC-AME	Controls	*p* value
N	28	27	–
Male, n (%)	11 (39.3%)	12 (44.4%)	0.6
Age (years)	51.1 [37.7–58.0]	40.7 [33.2–49.8]	0.14
BMI (kg/m^2^)	27.7 [26.1–30.1]	26.5 [24.6–28.9]	0.13
SBP (mm Hg)	141.0 [120.0–154.2]	115.7 [108.7–119.0]	< 0.0001*
DBP (mm Hg)	88.5 [78.7–97.5]	74.7 [70.0–75.7]	< 0.0001*
Serum cortisol (ug/dL) (nmol/L)	9.9 [8.6–12.5]	10.4 [7.4–16.7]	0.8
	273.1 [237.2–344.8]	286.9 [204.1–460.7]	
Serum cortisone (ug/dL) (nmol/L)	1.9 [1.8–2.1]	2.4 [2.1–2.8]	< 0.0001*
	52.7 [49.9–58.3]	66.6 [58.3–77.7]	
Serum F/E ratio	5.3 [4.7–6.1]	4.4 [3.7–5.9]	0.01*
PRA (ng/ml*h) (ng/L/s)	1.0 [0.7–1.8]	1.5 [1.3–2.0]	0.005*
	0.3 [0.2–0.5]	0.4 [0.4–0.6]	
Aldosterone (ng/dl) (pmol/L)	8.9 [5.4–13.6]	7.5 [5.6–11.7]	0.3
	246.9 [149.8–377.4]	208.1 [155.4–324.6]	
Serum Na (mEq/L)	141 [139–143]	140 [139–141]	0.2
Serum K (mEq/L)	4.1 [3.9–4.4]	4.3 [4.0–4.4]	0.4
FeNa (24 h %)	0.6 [0.4–0.8]	0.7 [0.6–0.9]	0.1
FEK (24 h %)	8.4 [6.3–10.7]	6.7 [5.7–8.3]	0.03*
Urinary Na (mEq/24 h)	124 [86–173]	141 [110–194]	0.2
Urinary K (mEq/24 h)	49.5 [38.0–62.8]	45.0 [27.0–59.0]	0.2
Urinary Na/K	2.4 [1.6–3.5]	3.5 [2.4–4.2]	0.004*

Values correspond to median [Q1–Q3].

*BMI* body mass index, *SBP* systolic blood pressure, *DBP* diastolic blood pressure, *F*/*E* cortisol to cortisone ratio, *PRA* plasma renin activity, *Na* sodium; *K*+ potassium, *FENa* fractional excretion of sodium, *FEK* fractional excretion of potassium.

*p < 0.05, Mann–Whitney test.

### NC-AME metabolomic profiling

To identify metabolite changes in NC-AME, we acquired untargeted metabolomic profiling of all serum samples in the discovery setup based on UPLC-Q-TOF/MS in both positive and negative ion modes. The data were obtained with the m/z, RT and data matrix of the peak area. In the positive and negative ion modes, we obtained 2798 and 3503 metabolites, respectively. The ion features of the QC samples were employed to calculate the relative standard deviation (RSD). As shown in Supplemental Fig. 1C, the 76.8% of the variables among 3503 ions acquired in ESI negative ion mode from the QC sample had RSD% less than 30%, and 86.1% of variables among the 2798 ions acquired in ESI positive ion mode from the QC sample had RSD% less than 30%. All these results suggested that the analytical method could be applied in metabolomics analysis with excellent repeatability and stability^30^ (Supplemental Fig. 3A/B).

### Clustering analyses of metabolites

To investigate the global metabolic variations, we first used PCA to analyze all samples acquired in both ion modes. The PCA plot (Supplemental Fig. 4A,B) presents an overview of all samples in the data and exhibits an unclear grouping trend between both groups (NC-AME and controls).

**Figure 1:**
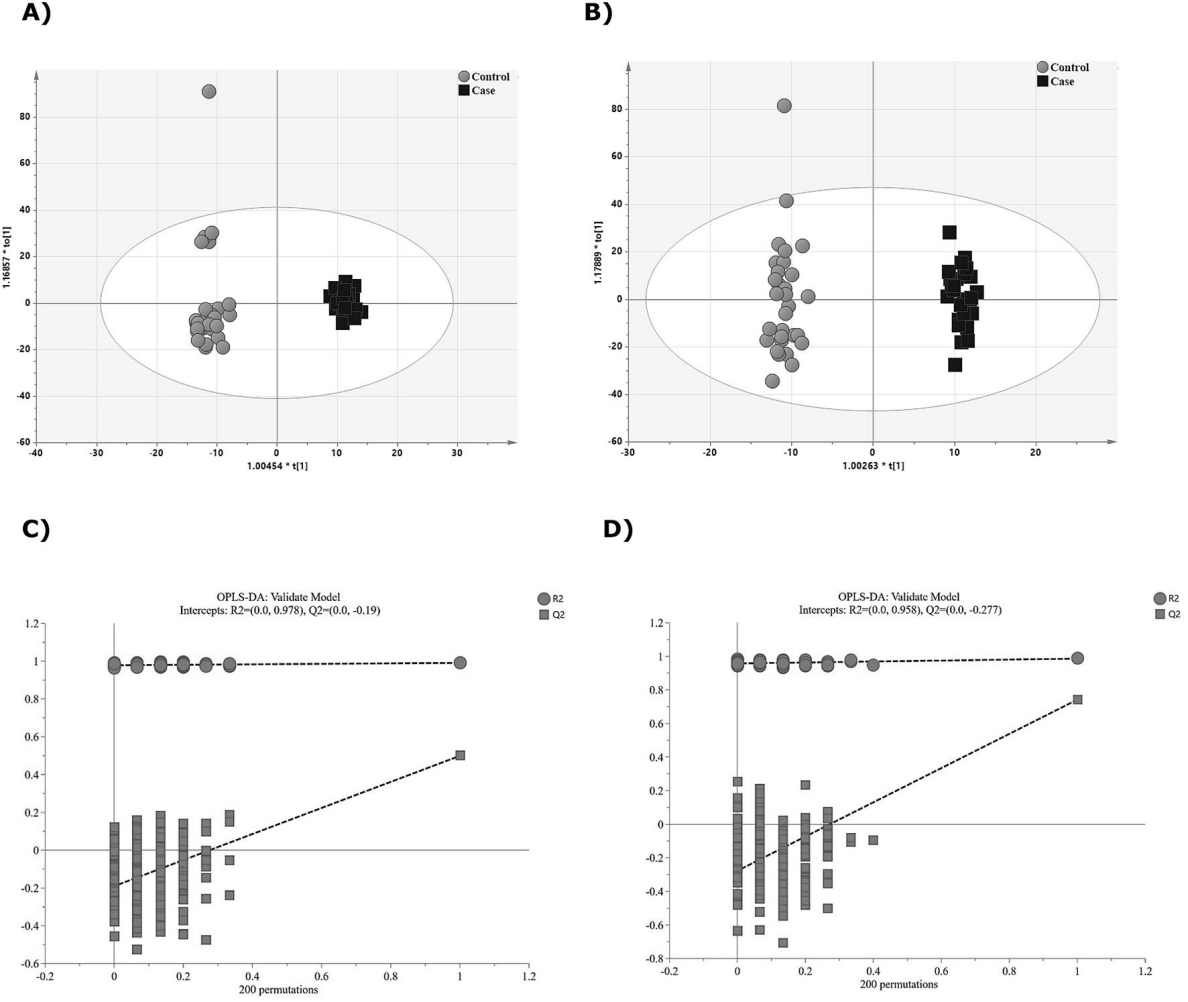
Discriminant analysis of groups NC-AME patients vs. control group. (**A**) OPLS-DA score scatter plots in ESI negative mode. X-axis and Y-axis represent score vectors summarizing all the variables entering the analysis. (**B**) Scatter plot of OPLS-DA in ESI positive data. (**C**) Validation plot for OPLS-DA model in ESI negative mode. A 200-time permutation test was used to assess the model. The y-axis intercepts were R2 (0.0, 0.978) and Q2 (0.0, − 0.19). (**D**) Validation plot for OPLS-DA model in ESI positive mode. The permutation tests were carried out with 200 random permutations. The y-axis intercepts were R2 (0.0, 0.958) and Q2 (0.0, − 0.277).

To obtain an improved separation and gain a better understanding of the variables responsible for the classification, a supervised clustering PLS-DA model analysis was applied. The score plot of PLS-DA analysis showed a distinct separation between the NC-AME and control groups (Supplemental Fig. 5A,B). The PLS-DA model in negative ion mode had an R2Y value of 0.99 and a Q2Y value of 0.815, and for model in positive ion mode had an R2Y of 0.90 and a Q2Y of 0.74 (Supplementary Table 1). To assess the model, a permutation test was applied (n = 200). The R2 and Q2 intercept values were 0.977 and 0.483 for negative ion mode, and 0.774 and 0.0389 for positive ion mode, respectively (Supplementary Fig. 5C,D). To refine the PLS-DA analysis, OPLS-DA analysis was performed to maximize the differences between groups in the model. OPLS-DA score plot (Fig. 1A,B) showed a clear separation of the control versus the NC-AME groups. The OPLS-DA model had an R2Y value of 0.991 (i.e., the model explained 99% of the variation observed within the data) and a Q2Y value of 0.499 for negative data, and an R2Y of 0.986 and Q2Y of 0.742 for positive data. Furthermore, a permutation test was applied. The R2 and Q2 intercept values were 0.978 and − 0.19 for negative ion mode (Fig. 1C), and 0.958 and − 0.277 for positive ion mode (Fig. 1D). We determined which differentially expressed metabolites have the most important role in separating the two groups. We obtained VIP values from the OPLS-DA analysis, differential metabolites were selected when the variable importance of the projection values were greater than 1.5 (VIP > 1.5) (Supplemental Figs. 6A/B and 7A/B).

**Figure 2:**
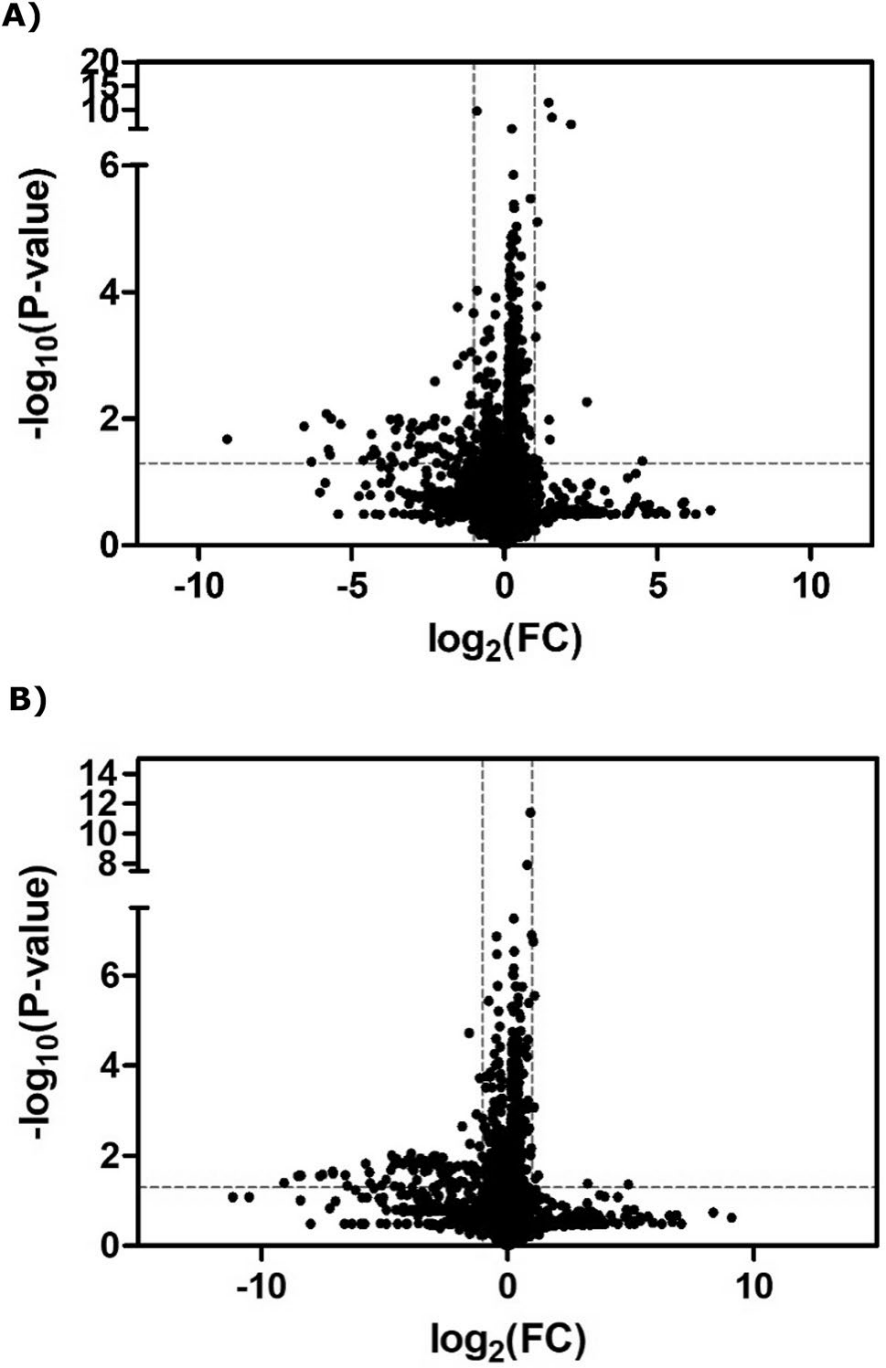
Volcano plot of differential abundance of metabolites found in metabolomics assay of NC-AME versus control subjects. The X-axis is the Log2 of metabolites levels (fold change) between subjects with NC-AME and control subjects. The Y-axis shows the − Log10(*p* value) obtained from comparison of control versus NCAME group by an unpaired student's t test. The range of Y > 1.30 (equal to *p* value of 0.05) and X > 1 were considered as significant increase; The range of Y > 1.30 and X < 1 were considered as significant decrease. (**A**) At negative ion mode (**B**) At positive ion mode.

**Figure 3:**
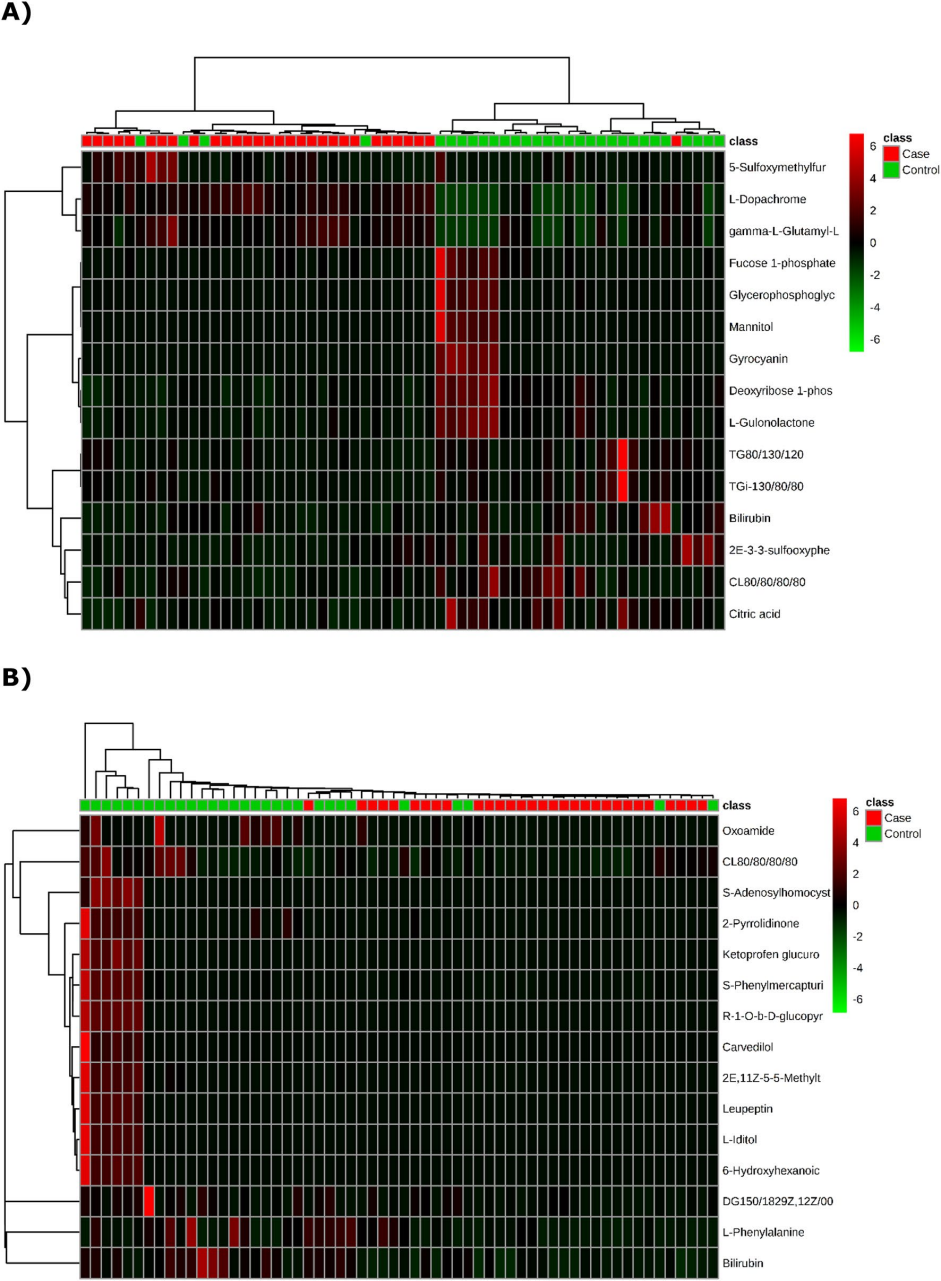
Hierarchical cluster analysis of metabolome data from significant metabolites. (**A**) Negative ion mode (**B**) Positive ion mode.

The significant differential metabolites of the NC-AME patients compared with the control subjects were visualized through volcano plots (Fig. 2) with further the false discovery rate (FDR) adjustment. The results showed that 21 (in the negative ion model) (Table 2) and 15 (in the positive ion model) (Table 3) differential metabolites distinguished the NC-AME from the healthy controls. Among these 36 metabolites from the NC-AME, three metabolites were significantly upregulated, and thirty-three were downregulated (Tables 2 and 3), compared with the respective metabolites from healthy controls. The heatmap plot of the differential metabolites in NC-AME versus control subjects is presented in Fig. 3, in which color intensity correlates with the degree of increase (red) and decrease (green) relative to the mean metabolite ratio.

**Table 2:** Identified metabolites in negative ion model related to NC-AME based on UPLC-Q-TOF/MS.

Retention time (min)	Molecular weight (g/mol)	Compound name	Formula	Pathway	Fold change	*p* value	q value
**Negative ion model**							
2.664	193.040	l-Dopachrome	C_9_H_7_NO_4_	Tyrosine metabolism	2.754	0.000	0.000
1.706	294.088	gamma-l-Glutamyl-l-methionine sulfoxide	C_10_H_18_N_2_O_6_S	–	2.162	0.000	0.000
4.798	192.026	Citric acid	C_6_H_8_O_7_	Transfer of acetyl groups into mitochondria Citric acid cycle Warburg effect	0.357	0.000	0.000
0.901	214.024	Deoxyribose 1-phosphate	C_5_H_11_O_7_P	Pentose phosphate pathway Pyrimidine metabolism Purine metabolism	0.475	0.000	0.000
0.898	178.047	l-Gulonolactone	C_6_H_10_O_6_	–	0.447	0.000	0.003
3.014	904.475	CL(8:0/8:0/8:0/8:0)	C_41_H_78_O_17_P_2_	–	0.381	0.005	0.003
4.045	584.263	Bilirubin	C_33_H_36_N_4_O_6_	Porphyrin metabolism	0.387	0.001	0.003
0.942	296.071	Gyrocyanin	C_17_H_12_O_5_	–	0.018	0.006	0.026
0.923	246.050	Glycerophosphoglycerol	C_6_H_15_O_8_P	–	0.111	0.014	0.037
0.935	244.034	Fucose 1-phosphate	C_6_H_13_O_8_P	Fructose and mannose degradation	0.362	0.015	0.037
0.891	182.078	Mannitol	C_6_H_14_O_6_	–	0.002	0.000	0.037
2.212	205.987	5-Sulfoxymethylfurfural	C_6_H_6_O_6_S	–	2.868	0.02	0.037
4.282	496.230	Glaucarubin	C_25_H_36_O_10_	–	0.135	0.04	0.040
11.369	540.438	TG(i-13:0/8:0/8:0)	C_32_H_60_O_6_	–	0.585	0.016	0.040
3.891	244.003	(2E)-3-[3-(sulfooxy)phenyl]prop-2-enoic acid	C_9_H_8_O_6_S	–	0.390	0.013	0.040
3.634	490.311	1-(13Z,16Z-docosadienoyl)-glycero-3-phosphate	C_25_H_47_O_7_P	–	0.017	0.022	0.040
12.411	596.501	TG(8:0/13:0/12:0)	C_36_H_68_O_6_	–	0.595	0.014	0.040
11.866	442.402	MG(24:0/0:0/0:0)	C_27_H_54_O_4_	–	0.544	0.016	0.040
3.138	372.105	Dihydroferulic acid 4-O-glucuronide	C_16_H_20_O_10_	–	0.442	0.035	0.048
3.65	428.310	Glyceryl lactooleate	C_24_H_44_O_6_	–	0.073	0.036	0.048
4.486	286.189	Androstenedione	C_19_H_26_O_2_	Androstenedione metabolism Androgen and estrogen metabolism	0.011	0.034	0.048

Q-values are the adjusted *p* values using an optimized FDR approach.

**Table 3:** Identified metabolites in positive ion model related to NC-AME based on UPLC-Q-TOF/MS.

Retention time (min)	Molecular weight (g/mol)	Compound name	Formula	Pathway	Fold change	*p* value	q value
**Positive ion model**							
4.047	584.265	Bilirubin	C_33_H_36_N_4_O_6_	Porphyrin metabolism	0.339	0.000	0.000
3.011	904.478	CL(8:0/8:0/8:0/8:0)	C_41_H_78_O_17_P_2_	–	0.417	0.001	0.027
2.822	239.062	S-Phenylmercapturic acid	C_11_H_13_NO_3_S	–	0.346	0.005	0.030
1.292	85.053	2-Pyrrolidinone	C_4_H_7_NO	–	0.086	0.012	0.008
5.989	308.184	(R)-1-O-b-d-glucopyranosyl-1,3-octanediol	C_14_H_28_O_7_	–	0.061	0.012	0.025
0.883	384.125	S-Adenosylhomocysteine	C_14_H_20_N_6_O_5_S	Methionine metabolism Histidine metabolism Glycine and serine metabolism Tryptophan metabolism Tyrosine metabolism Arginine and proline metabolism Methylhistidine metabolism Betaine metabolism Carnitine synthesis Nicotinate and nicotinamide metabolism Estrone metabolism Phosphatidylcholine biosynthesis Catecholamine biosynthesis Ubiquinone biosynthesis	0.095	0.013	0.027
2.833	430.123	Ketoprofen glucuronide	C_22_H_22_O_9_	–	0.151	0.013	0.027
0.905	232.056	(2E,11Z)-5-[5-(Methylthio)-4-penten-2-ynyl]-2-furanacrolein	C_13_H_12_O_2_S	–	0.096	0.015	0.027
1.018	192.090	Oxoamide	C_10_H_12_N_2_O_2_	–	0.361	0.016	0.027
0.901	165.077	l-Phenylalanine	C_9_H_11_NO_2_	Phenylalanine and tyrosine metabolism	0.426	0.021	0.027
5.988	132.079	6-Hydroxyhexanoic acid	C_6_H_12_O_3_	–	0.152	0.024	0.030
3.629	426.296	Leupeptin	C_20_H_38_N_6_O_4_	–	0.007	0.025	0.030
12.241	578.491	DG(15:0/18:2(9Z,12Z)/0:0)	C_36_H_66_O_5_	–	0.391	0.027	0.030
0.9	182.079	l-Iditol	C_6_H_14_O_6_	–	0.003	0.028	0.030
4.48	406.190	Carvedilol	C_24_H_26_N_2_O_4_	–	0.140	0.045	0.045

Q-values are the adjusted *p* values using an optimized FDR approach.

### Enrichment and clustering of metabolites of interest

To identify the relationships of the metabolites, we constructed a correlation network diagram based on the KEGG databases and MBRole. Under the limiting condition of *p* < 0.05 in the MBRole, there are primarily 25 enriched metabolic pathways, including 36 highlighted metabolites, that were involved, while the methylhistidine metabolism and transfer of acetyl groups into mitochondria pathway ranked highest, which provides key information for constructing a metabolomic network diagram (Supplemental Fig. 8A/B).

### Prediction regression model

**Figure 4:**
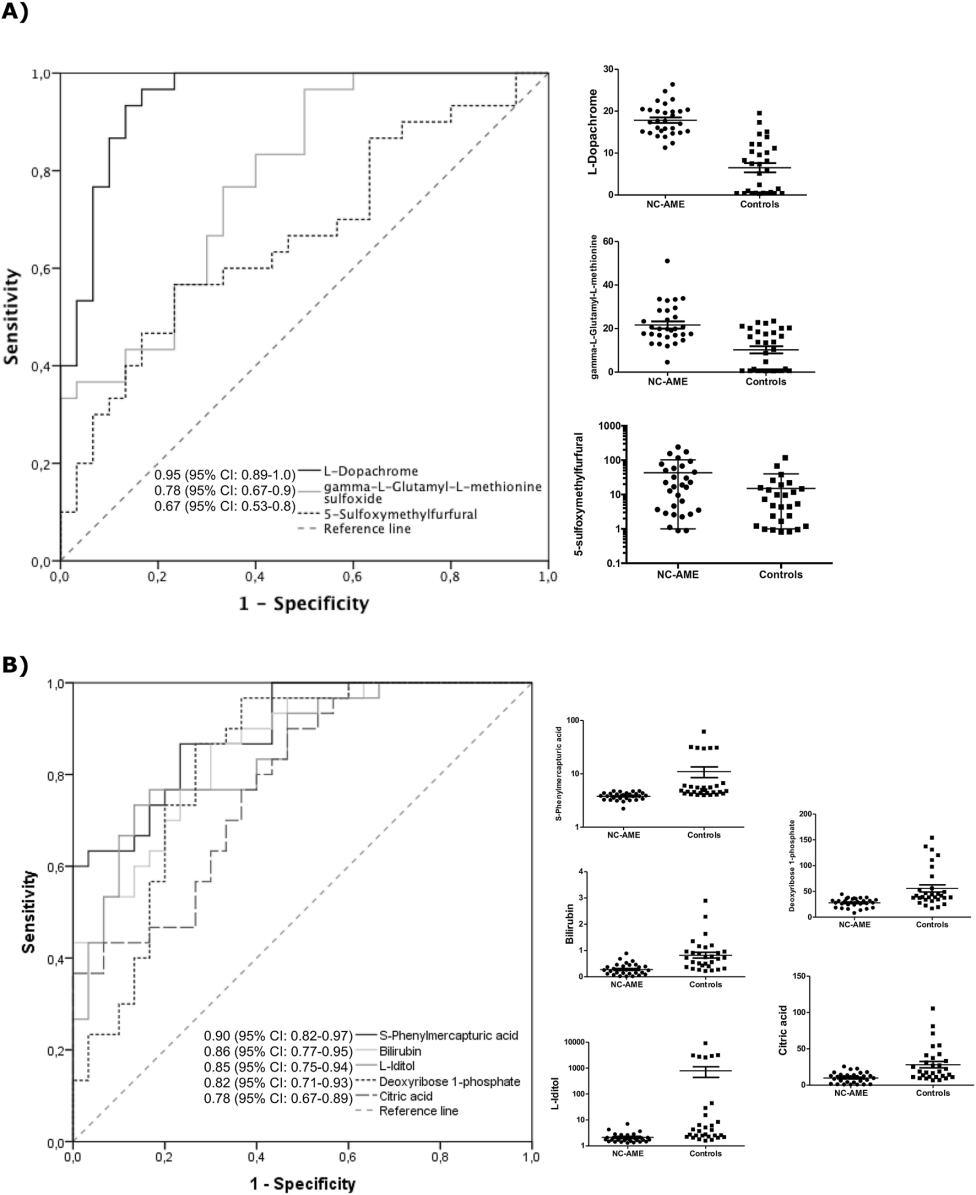
Evaluation of diagnostic efficacy using ROC curve for significant top 8 metabolites in serum differentially expressed in NC-AME and control subjects, as detected by UPLC–TOF–MS. (**A**) Upregulated metabolites; (**B**) Downregulated metabolites.

To assess the diagnostic capacity of the significant metabolites in the identification of the NC-AME phenotype, we applied ROC curve analysis to the data, which allowed to identify 8 metabolites with an AUC greater than 0.650. The area under the ROC curve for the upregulated metabolites were l-dopachrome, gamma-l-glutamyl-l-methionine sulfoxide and 5-sulfoxymethylfurfural (SMF), was 0.95 (95% CI 0.89–1.0), 0.78 (95% CI 0.67–0.9), 0.67 (95% CI 0.53–0.8) respectively, in NC-AME subjects (Fig. 4A). The area under the ROC curve for downregulated metabolites, S-phenylmercapturic acid (SPMA), bilirubin, l-iditol, deoxyribose 1-phosphate, and citric acid, was 0.9 (95% CI 0.82–0.97), 0.86 (95% CI 0.77–0.95), 0.85 (95% CI 0.75–0.94), 0.82 (95% CI 0.71–0.93), and 0.78 (95% CI 0.67–0.89) in NC-AME subjects, respectively (Fig. 4B).

To test the predictive value of these 8 metabolites we performed a logistic regression model with a ten fold nested cross-validation, which showed that all these metabolites are able to discriminate between the NC-AME and control subjects with an accuracy of 0.92 (95% CI 0.83–1.00), a sensitivity of 0.93 (95% CI 0.81–1.00), a specificity of 0.90 (95% CI 0.77–1.00) and a AUC of 0.92 (95% CI 0.82–1.00).

Since low serum cortisone has been reported to be one of the best predictors of MR activation in NC-AME subjects^9^, we performed linear regression analyses between serum cortisone and significant metabolites, adjusted for age, BMI, SBP and DBP we identified a negative association of cortisone with gamma-l-glutamyl-l-methionine sulfoxide (r = − 0.29; *p* = 0.039) and 5-sulfoxymethylfurfural (r = − 0.39; *p* = 0.003).

## Discussion

This is the first report to describe a metabolomic study in NC-AME patients. The discovery study was based on an untargeted metabolomic analysis of serum samples from NC-AME patients and identified 36 differentially regulated metabolites, specifically 3 upregulated metabolites and 33 downregulated metabolites. For these 36 metabolites, we evaluated their diagnostic capacity as biomarkers for this phenotype. We observed that l-dopachrome and SPMA had the highest sensitivity and specificity to discriminate the NC-AME condition, followed by bilirubin, l-iditol, deoxyribose 1-phosphate, citric acid, gamma-l-glutamyl-l-methionine sulfoxide and 5-sulfoxymethylfurfural (SMF). The combination of these eight metabolites showed a high sensitivity (93%) and specificity (90%) to discriminate for NC-AME from control subjects.

The metabolites gamma-l-glutamyl-l-methionine sulfoxide, l-dopachrome and SMF were significantly increased in NC-AME patients (Table 2; Fig. 4). The gamma-l-glutamyl-l-methionine is an organic compound that belongs to the class of dipeptides and is generated in conditions of oxidative stress^31,32^. Methionine sulfoxide has been proposed as a physiological marker of oxidative stress, which is a key mechanism of endothelial dysfunction, as observed in NC-AME subjects and even more notably in hypertensive patients^33^. Recently, Zhao and colleagues in a urine metabolomic study revealed the involvement of oxidative stress metabolic pathways and amino acid metabolism in essential hypertension^34^. Additional evidence suggested that essential hypertensives (EH) may be a disorder of inherited amino acid metabolism^35^. However, the increased levels of these two metabolites have never been explored in NC-AME. Similarly, l-dopachrome belongs to the class of organic compounds known as l-alpha-amino acids, and elevated levels of this metabolite indicate an increase in tyrosine metabolism, which includes the biosynthesis of melanin^36,37^. In this way, gamma-l-glutamyl-l-methionine, l-dopachrome or metabolites associated with this metabolic pathway should be further evaluated as endogenous inhibitors of 11β-HSD2, and further research is required to reveal such effects.

Along with the findings described above, we also observed that subjects with NC-AME have high levels of the organic compound SMF, which is also negatively associated with serum cortisone. SMF is generated from the metabolism of 5-hydroxymethylfurfural, a reactive metabolite that can bind to DNA and cause mutagenic effects^38^. SMF is toxic, since it accumulates in kidney proximal tubules by improper excretion due to renal reabsorption processes^39^, which leads to the above mentioned damage to DNA and proteins. In addition, we show that SPMA has a good diagnostic ability to identify this phenotype of NC-AME. SPMA belongs to the family of *N*-acyl-alpha amino acids and derivatives, and is a benzene metabolite that is catalyzed by glutathione S-transferases and has been considered a biomarker of oxidative damage. These findings may support the complementary use of biomarkers associated with oxidative stress and renal damage, such as microalbuminuria, in NC-AME subjects.

On the other hand, bilirubin also has a good discriminatory capacity to identify the NC-AME phenotype and is decreased in these subjects. Bilirubin has previously been characterized as an antioxidative and anti-inflammatory protective factor with respect to peripheral vascular diseases^40,41^, suggesting that NC-AME subjects with lower bilirubin levels may have lower protective antioxidant effects. Although various studies have shown some association of high bilirubin with a low incidence of hypertension, the present findings should be viewed with caution and should be further evaluated^42^.

Other metabolites that are decreased in NC-AME, such as l-iditol and deoxyribose 1-phosphate, also have a good discriminatory capacity to identify the NC-AME phenotype. l-Iditol is a sugar alcohol and is part of various metabolic reactions in organisms that include fructose and mannose metabolism. On the other hand, deoxyribose 1-phosphate is involved in pentose phosphate pathways (PPPs). PPP is a multienzyme pathway that shares a common starting molecule with glycolysis, glucose-6-phosphate. PPP plays a critical role in regulating cell growth by supplying cells with not only ribose-5-phosphate but also NADPH for detoxification of intracellular ROS, reductive biosynthesis, and ribose biogenesis. Thus, the PPP can adapt to the needs of a particular cell at a time point when a change in the metabolism of a cell is required. However, these mechanisms may be affected in subjects with NC-AME.

In this report, we found low levels of citric acid in NC-AME subjects. The citric acid cycle (CAC) provides precursors of certain amino acids, as well as the reducing agent NADH, that are used in numerous reactions. Regarding 11β-HSD2 enzymatic activity, it is known that this enzyme is highly dependent on cofactor NAD^+^^6^, which is essential for proper cortisol catabolic activity in the kidney and other nonepithelial tissues. Thus, a decrease in the activity of the citric acid cycle should affect the synthesis of NAD+, which is a critical cofactor for the 11β-HSD2 enzyme. The diminished CAC activity may be due to a reduction in overall mitochondrial biogenesis, reduced expression of the genes encoding citric acid enzymes, or reduced citric acid cycle substrate availability.

We observed a decrease in *S*-adenosyl-l-homocysteine (SAH), which is the metabolic precursor of homocysteine and is a negative regulator of most cell methyltransferases associated with DNA hypermethylation^43^. Low SAH levels observed in NC-AME subjects are indicated to be associated with higher expression of DNA-methyltransferase and hypermethylation of the HSD11B2 promoter^44^, which is expected to decrease HSD1B2 expression and subsequently affect cortisol to cortisone metabolism in these subjects. Recently, Lana et al. in a similar untargeted metabolomics analysis in EH^45^, also detected a dysregulation in the urine levels of sulfur-containing metabolites (thiocysteine and homomethionine), purines (SAH, AMP, allantoate, and hydroxyisourate) and pyrimidines (dihydrothymine, uracil, and UDP), among others^45^, suggesting that NC-AME may be associated with impaired sulfur-containing metabolites, such as SAH.

Various publications^17,20^ have addressed the effects of the inhibition of 11β-HSD2 by endogenous and exogenous inhibitors. In this study, we identified also the presence of some of these endogenous inhibitors, such as cholic acid derivatives, and exogenous inhibitors, such as perfluorohexane sulfonic acid, perfluorooctanesulfonic acid, perfluorooctanoic acid, diethyl-phthalic acid, and monoethylphthalate. However, we did not observe significant differences between the subjects with NC-AME and the healthy controls (data not shown).

Regarding the regression modeling to select and validate the marker metabolites able to discriminate NC-AME from control subjects, we performed a logistic regression and ROC curve analyses. We found 8 metabolites including l-dopachrome, gamma-l-glutamyl-l-methionine sulfoxide, 5-sulfoxymethylfurfural (SMF), S-phenylmercapturic acid (SPMA), bilirubin, l-iditol, deoxyribose 1-phosphate and citric acid, which contributed to the combined model and achieved the highest AUC value corresponding to 92%. These biomarkers have good diagnostic accuracy in distinguishing NC-AME from controls subjects with and accuracy of 92%, a sensitivity of 93% and a specificity of 90%. Moreover, two of them, gamma-l-glutamyl-l-methionine sulfoxide and SMF showed significant association with cortisone, the principal analyte associated to the NC-AME phenotype, using a linear regression model adjusted by age, BMI, and blood pressure. However, we are aware this is a discovery study, which should be further evaluated in another cohort of subjects to validate these metabolites and confirm these findings.

In summary, we found by a metabolomic study 8 metabolites that are potentially useful as biomarkers associated with the NC-AME phenotype. Moreover, these metabolites and their respective metabolic pathways should help to identify the pathophysiological mechanism governing this condition. Here, we encourage future in vitro and in vivo assays involving these metabolites in order to define their roles in 11β-HSD2 expression and activity. Beside this knowledge, is necessary to advance in the development of dedicated assays for single or grouped metabolites in order to support novel algorithms for identification of NC-AME subjects.

## Supplementary Information

Supplementary Information.

## References

[1] Yau M (2017). Clinical, genetic, and structural basis of apparent mineralocorticoid excess due to 11beta-hydroxysteroid dehydrogenase type 2 deficiency. Proc. Natl. Acad. Sci. U.S.A..

[2] Arriza JL (1987). Cloning of human mineralocorticoid receptor complementary DNA: Structural and functional kinship with the glucocorticoid receptor. Science.

[3] Arriza JL, Simerly RB, Swanson LW, Evans RM (1988). The neuronal mineralocorticoid receptor as a mediator of glucocorticoid response. Neuron.

[4] Ferrari P, Lovati E, Frey FJ (2000). The role of the 11beta-hydroxysteroid dehydrogenase type 2 in human hypertension. J. Hypertens..

[5] Carvajal CA, Tapia-Castillo A, Vecchiola A, Baudrand R, Fardella CE (2020). Classic and nonclassic apparent mineralocorticoid excess syndrome. J. Clin. Endocrinol. Metab..

[6] Carvajal CA (2003). Two homozygous mutations in the 11 beta-hydroxysteroid dehydrogenase type 2 gene in a case of apparent mineralocorticoid excess. J. Clin. Endocrinol. Metab..

[7] Carvajal CA (2018). Serum cortisol and cortisone as potential biomarkers of partial 11beta-hydroxysteroid dehydrogenase type 2 deficiency. Am. J. Hypertens..

[8] Mune T, Rogerson FM, Nikkila H, Agarwal AK, White PC (1995). Human hypertension caused by mutations in the kidney isozyme of 11 beta-hydroxysteroid dehydrogenase. Nat. Genet..

[9] Tapia-Castillo A (2019). Clinical, biochemical, and genetic characteristics of "nonclassic" apparent mineralocorticoid excess syndrome. J. Clin. Endocrinol. Metab..

[10] Baudrand R, Vaidya A (2018). The low-renin hypertension phenotype: Genetics and the role of the mineralocorticoid receptor. Int. J. Mol. Sci..

[11] Baudrand R (2017). Continuum of renin-independent aldosteronism in normotension. Hypertension.

[12] Brown JM (2017). The spectrum of subclinical primary aldosteronism and incident hypertension: A cohort study. Ann. Intern. Med..

[13] Tapia-Castillo A (2019). Downregulation of exosomal miR-192-5p and miR-204-5p in subjects with nonclassic apparent mineralocorticoid excess. J. Transl. Med..

[14] Morris DJ (1992). Detection of glycyrrhetinic acid-like factors (GALFs) in human urine. Hypertension.

[15] Morris DJ, Latif SA, Hardy MP, Brem AS (2007). Endogenous inhibitors (GALFs) of 11beta-hydroxysteroid dehydrogenase isoforms 1 and 2: Derivatives of adrenally produced corticosterone and cortisol. J. Steroid Biochem. Mol. Biol..

[16] Latif SA, Sheff MF, Ribeiro CE, Morris DJ (1997). Selective inhibition of sheep kidney 11 beta-hydroxysteroid dehydrogenase isoform 2 activity by 5 alpha-reduced (but not 5 beta) derivatives of adrenocorticosteroids. Steroids.

[17] Ma X, Lian QQ, Dong Q, Ge RS (2011). Environmental inhibitors of 11beta-hydroxysteroid dehydrogenase type 2. Toxicology.

[18] Latif SA, Conca TJ, Morris DJ (1990). The effects of the licorice derivative, glycyrrhetinic acid, on hepatic 3 alpha- and 3 beta-hydroxysteroid dehydrogenases and 5 alpha- and 5 beta-reductase pathways of metabolism of aldosterone in male rats. Steroids.

[19] Kumagai A, Yano S, Otomo M (1957). Study on the corticoid-like action of glycyrrhizine and the mechanism of its action. Endocrinol. Jpn..

[20] Zhou C, Ye F, Wu H, Ye H, Chen Q (2017). Recent advances in the study of 11beta-hydroxysteroid dehydrogenase type 2 (11beta-HSD2)inhibitors. Environ. Toxicol. Pharmacol..

[21] Patti GJ, Yanes O, Siuzdak G (2012). Innovation: Metabolomics: The apogee of the omics trilogy. Nat. Rev. Mol. Cell Biol..

[22] Wawrzyniak R (2019). Untargeted metabolomics provides insight into the mechanisms underlying resistant hypertension. Curr. Med. Chem..

[23] Mulatero P (2002). Drug effects on aldosterone/plasma renin activity ratio in primary aldosteronism. Hypertension.

[24] Montero J, Soto J, Fardella C, Foradori A, Valdes G (1998). Measurement of low levels of plasma renin activity. A methodological improvement. Rev. Med. Chile.

[25] Whelton PK (2017). ACC/AHA/AAPA/ABC/ACPM/AGS/APhA/ASH/ASPC/NMA/PCNA guideline for the prevention, detection, evaluation, and management of high blood pressure in adults: A report of the American College of Cardiology/American Heart Association Task Force on clinical practice guidelines. J. Am. Coll. Cardiol..

[26] Liu X (2015). Discovery and validation of plasma biomarkers for major depressive disorder classification based on liquid chromatography-mass spectrometry. J. Proteome Res..

[27] Chen C (2020). Metabolomics reveals metabolite changes of patients with pulmonary arterial hypertension in China. J. Cell. Mol. Med..

[28] Zhao JH (2019). Circulating plasma metabolomic profiles differentiate rodent models of pulmonary hypertension and idiopathic pulmonary arterial hypertension patients. Am. J Hypertens..

[29] Pedregosa F, Varoquaux G, Gramfort A, Michel V, Thirion B, Grisel O, Blondel M, Prettenhofer P, Weiss R, Dubourg V, Vanderplas J (2011). Scikit-learn: Machine learning in python. J. Mach. Learn. Res..

[30] Broadhurst D (2018). Guidelines and considerations for the use of system suitability and quality control samples in mass spectrometry assays applied in untargeted clinical metabolomic studies. Metab. Off. J. Metab. Soc..

[31] Cabreiro F, Picot CR, Friguet B, Petropoulos I (2006). Methionine sulfoxide reductases: Relevance to aging and protection against oxidative stress. Ann. N. Y. Acad. Sci..

[32] Picot CR (2006). Alterations in mitochondrial and cytosolic methionine sulfoxide reductase activity during cardiac ischemia and reperfusion. Exp. Gerontol..

[33] Guzik TJ, Touyz RM (2017). Oxidative stress, inflammation, and vascular aging in hypertension. Hypertension.

[34] Zhao H (2018). Identification of essential hypertension biomarkers in human urine by non-targeted metabolomics based on UPLC-Q-TOF/MS. Clin. Chim. Acta Int. J. Clin. Chem..

[35] Min X, Lee BH, Cobb MH, Goldsmith EJ (2004). Crystal structure of the kinase domain of WNK1, a kinase that causes a hereditary form of hypertension. Structure.

[36] Tsukamoto K, Jackson IJ, Urabe K, Montague PM, Hearing VJ (1992). A second tyrosinase-related protein, TRP-2, is a melanogenic enzyme termed DOPAchrome tautomerase. EMBO J..

[37] Leonard LJ, Townsend D, King RA (1988). Function of dopachrome oxidoreductase and metal ions in dopachrome conversion in the eumelanin pathway. Biochemistry.

[38] Monien BH, Engst W, Barknowitz G, Seidel A, Glatt H (2012). Mutagenicity of 5-hydroxymethylfurfural in V79 cells expressing human SULT1A1: Identification and mass spectrometric quantification of DNA adducts formed. Chem. Res. Toxicol..

[39] Bakhiya N, Monien B, Frank H, Seidel A, Glatt H (2009). Renal organic anion transporters OAT1 and OAT3 mediate the cellular accumulation of 5-sulfooxymethylfurfural, a reactive, nephrotoxic metabolite of the Maillard product 5-hydroxymethylfurfural. Biochem. Pharmacol..

[40] Djousse L (2001). Total serum bilirubin and risk of cardiovascular disease in the Framingham offspring study. Am. J. Cardiol..

[41] Kunutsor SK, Bakker SJ, Gansevoort RT, Chowdhury R, Dullaart RP (2015). Circulating total bilirubin and risk of incident cardiovascular disease in the general population. Arterioscler. Thromb. Vasc. Biol..

[42] Chin HJ (2009). The bilirubin level is negatively correlated with the incidence of hypertension in normotensive Korean population. J. Korean Med. Sci..

[43] James SJ, Melnyk S, Pogribna M, Pogribny IP, Caudill MA (2002). Elevation in S-adenosylhomocysteine and DNA hypomethylation: Potential epigenetic mechanism for homocysteine-related pathology. J. Nutr..

[44] Pizzolo F (2015). Apparent mineralocorticoid excess by a novel mutation and epigenetic modulation by HSD11B2 promoter methylation. J. Clin. Endocrinol. Metab..

[45] Lana A (2019). Urinary metabolic signature of primary aldosteronism: Gender and subtype-specific alterations. Proteom. Clin. Appl..

