# Supplementary material for: Novel metabolomic profile of subjects with non-classic apparent mineralocorticoid excess
Source: Sci Rep. 2021 Aug 25;11:17156. doi: 10.1038/s41598-021-96628-6 (PMC8387493; doi:10.1038/s41598-021-96628-6)
Supplement: Supplementary file 1 — Supplementary Information. [file 41598_2021_96628_MOESM1_ESM.pdf]

NOVEL METABOLOMIC PROFILE OF SUBJECTS WITH NON-CLASSIC APPARENT MINERALOCORTICOID EXCESS

Supplementary Material

Alejandra Tapia-Castillo*[a,b,c], Cristian A. Carvajal*[a,b,c], Xaviera López-Cortés[d], Andrea Vecchiola[a,b,c], *Carlos E. Fardella[a,b,c]

[a]Department of Endocrinology, School of Medicine, Pontificia Universidad Católica de Chile, Santiago, Chile. [b]Millennium Institute on Immunology and Immunotherapy (IMII-ICM), Santiago, Chile. [c]Centro Traslacional de Endocrinología UC (CETREN-UC). [d]Department of Computer Science and Industries, Faculty of Engineering Science, Universidad Católica del Maule, Talca, Chile.

*contributed equally to this work

Running head: Metabolomic profile of NC-AME subjects

Keywords: metabolomics, nonclassic AME, mineralocorticoid receptor, hypertension

***Correspondence and reprint requests:**

Carlos E. Fardella, MD

Departamento de Endocrinología, Escuela de Medicina

Pontificia Universidad Católica de Chile

Diagonal Paraguay 362, piso 4

Santiago 8330077, CHILE

Ph: (56-2) 2354-3634

cfardella@med.puc.cl

Supplementary Table 1: Model quality and description

Parameter	Model	Type	A	N	R2X(cum)	R2Y(cum)	Q2(cum)
**Negative**	M1	PCA-X	6	55	0.334	/	0.0835
	M2	PLS-DA	4	55	0.232	0.991	0.815
	M3	OPLS-DA	1+3+0	55	0.232	0.991	0.499
**Positive**	M1	PCA-X	10	55	0.482	/	0.0847
	M2	PLS-DA	2	55	0.148	0.901	0.74
	M3	OPLS-DA	1+3+0	55	0.233	0.986	0.742

Note: In PCA analysis, R2X> 0.4 is better; In PLS-DA and OPLS-DA analysis, the R2X parameter is not important, mainly R2Y and Q2. The two values> 0.5 are better, the closer to 1, the better. Cum means cumulative. A: The component number.

A)

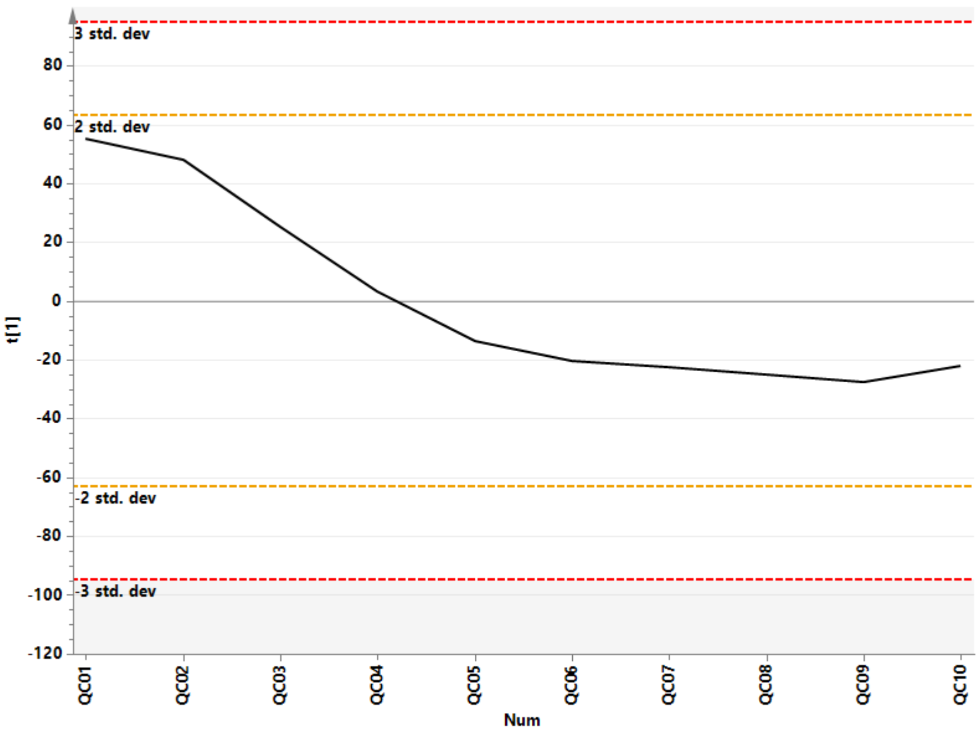

3 std. dev
2 std. dev
-2 std. dev
-3 std. dev
80
60
40
20
0
-20
-40
-60
-80
-100
-120
t[1]
QC01
QC02
QC03
QC04
QC05
QC06
QC07
QC08
QC09
QC10
Num

B)

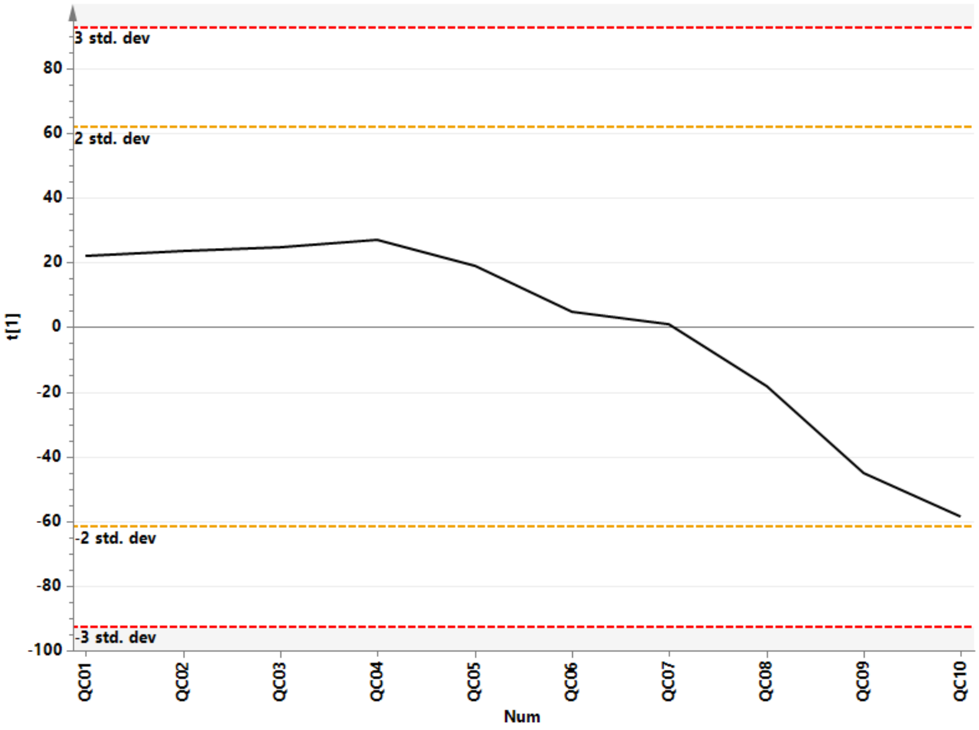

3 std. dev
2 std. dev
-2 std. dev
-3 std. dev
80
60
40
20
0
-20
-40
-60
-80
-100
t[1]
QC01
QC02
QC03
QC04
QC05
QC06
QC07
QC08
QC09
QC10
Num

C)

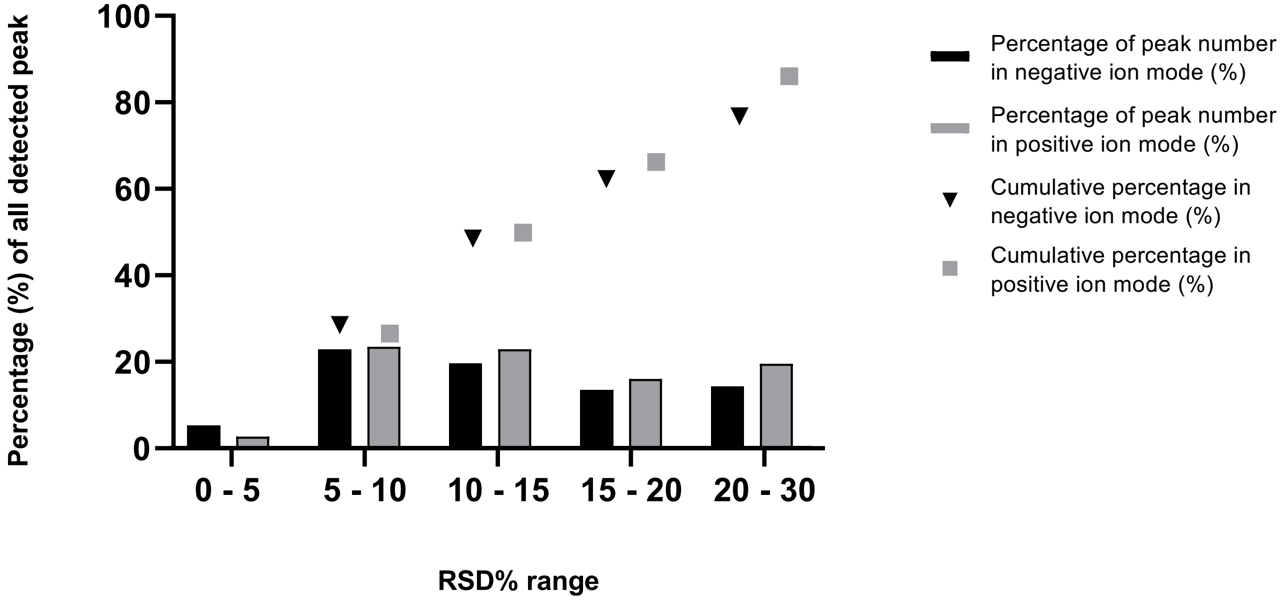

Supplementary Figure 1. The PCA score plot of the QC samples, the X axis indicates the number of QC samples, the Y axis indicates the range of RSD. A) negative mode; B) positive mode. C) Relative standard deviation (RSD%) distribution of all metabolites in the pooled quality control (QC) samples.

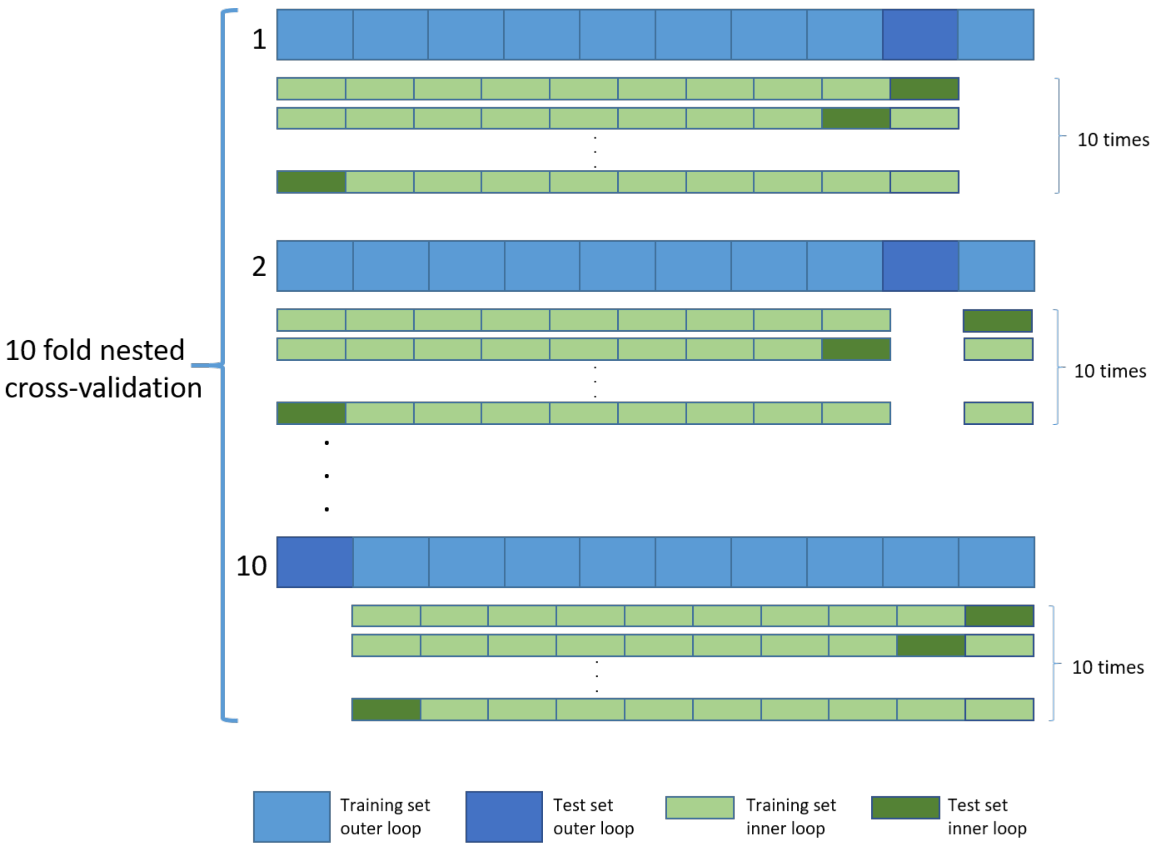

Supplementary Figure 2. Scheme of 10 fold nested cross-validation.

A)

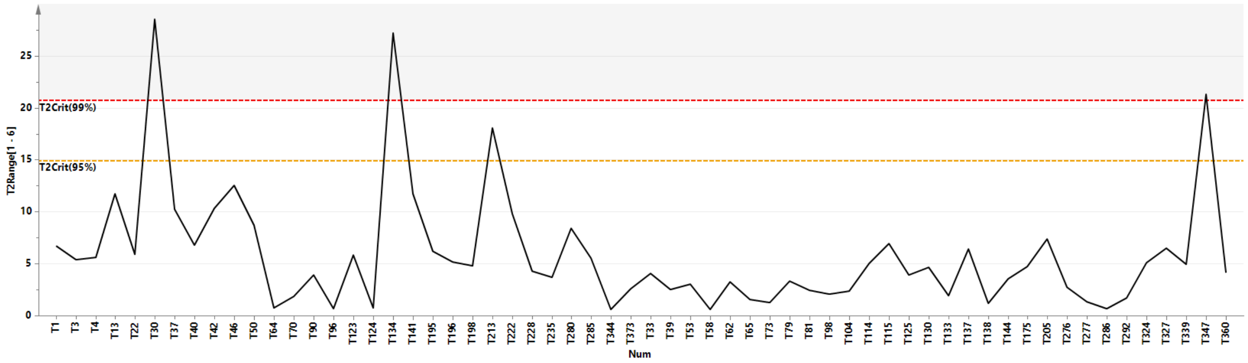

B)

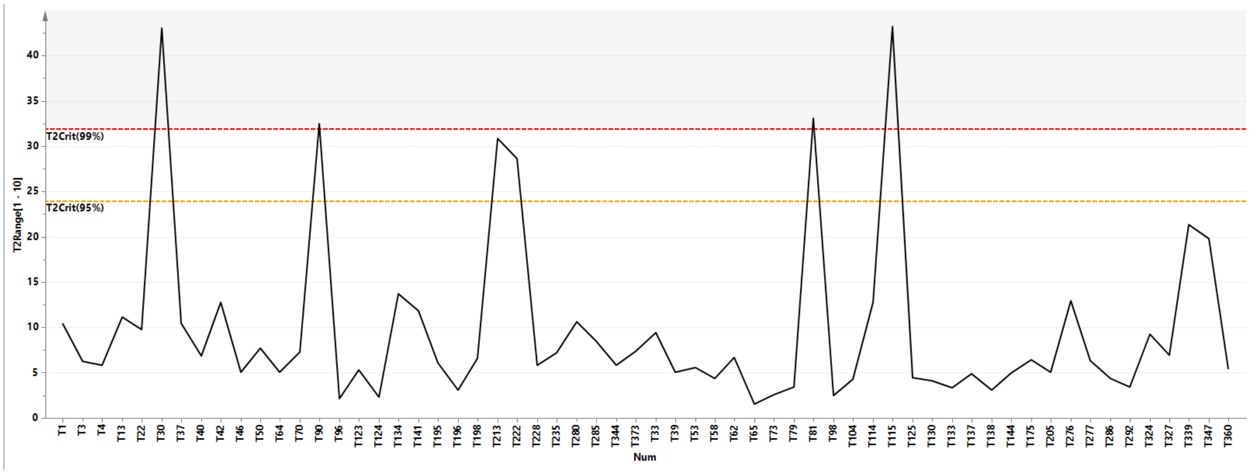

Supplementary Figure. 3 The line plot of samples, the X axis indicates the number of samples, the Y axis indicates the 95% confidence interval. A) ESI-mode; B) ESI+mode

A)

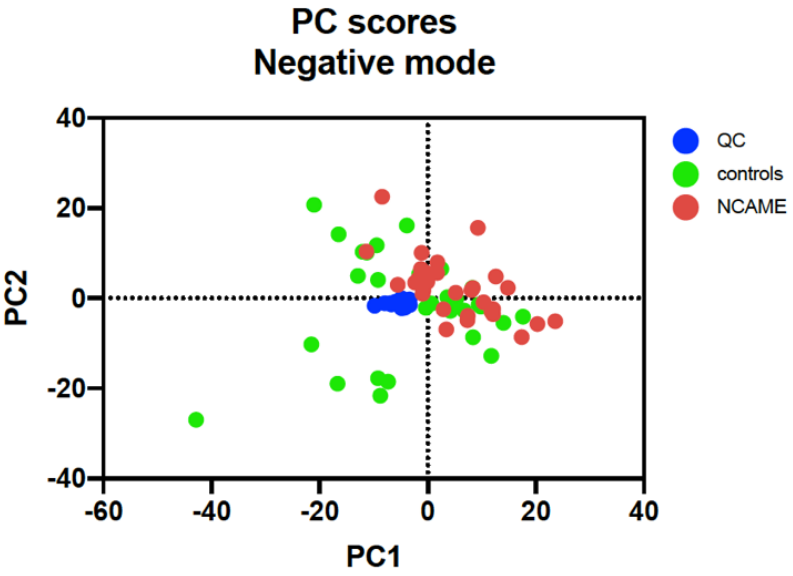

B)

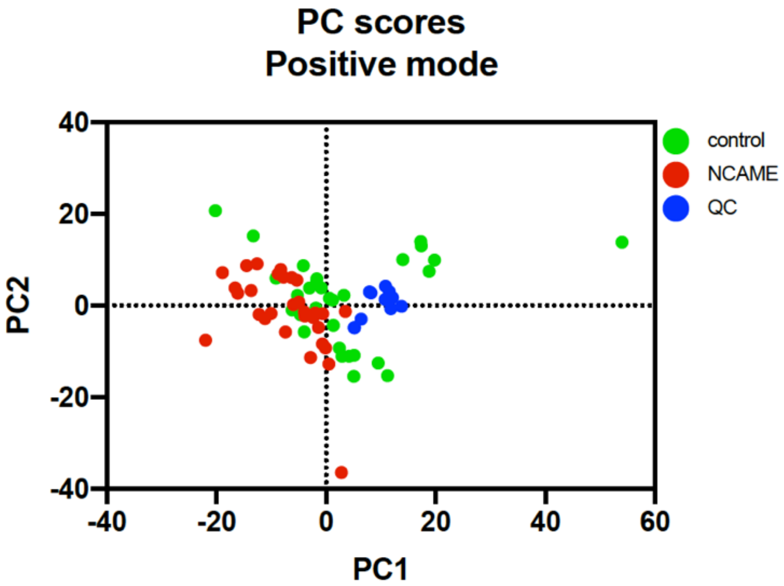

Supplementary Figure 4. The scores scatter plot of Principal component analysis (PCA) model based in untargeted metabolomic profiling of serum sample from NCA-ME and control subjects. On a PCA score plot each dot represents a sample. **A)** show negative electrospray ionization (ESI) mode **B)** show positive electrospray ionization (ESI) mode. PC1, first principle component score; PC2, second principle component score; QC, quality control sample.

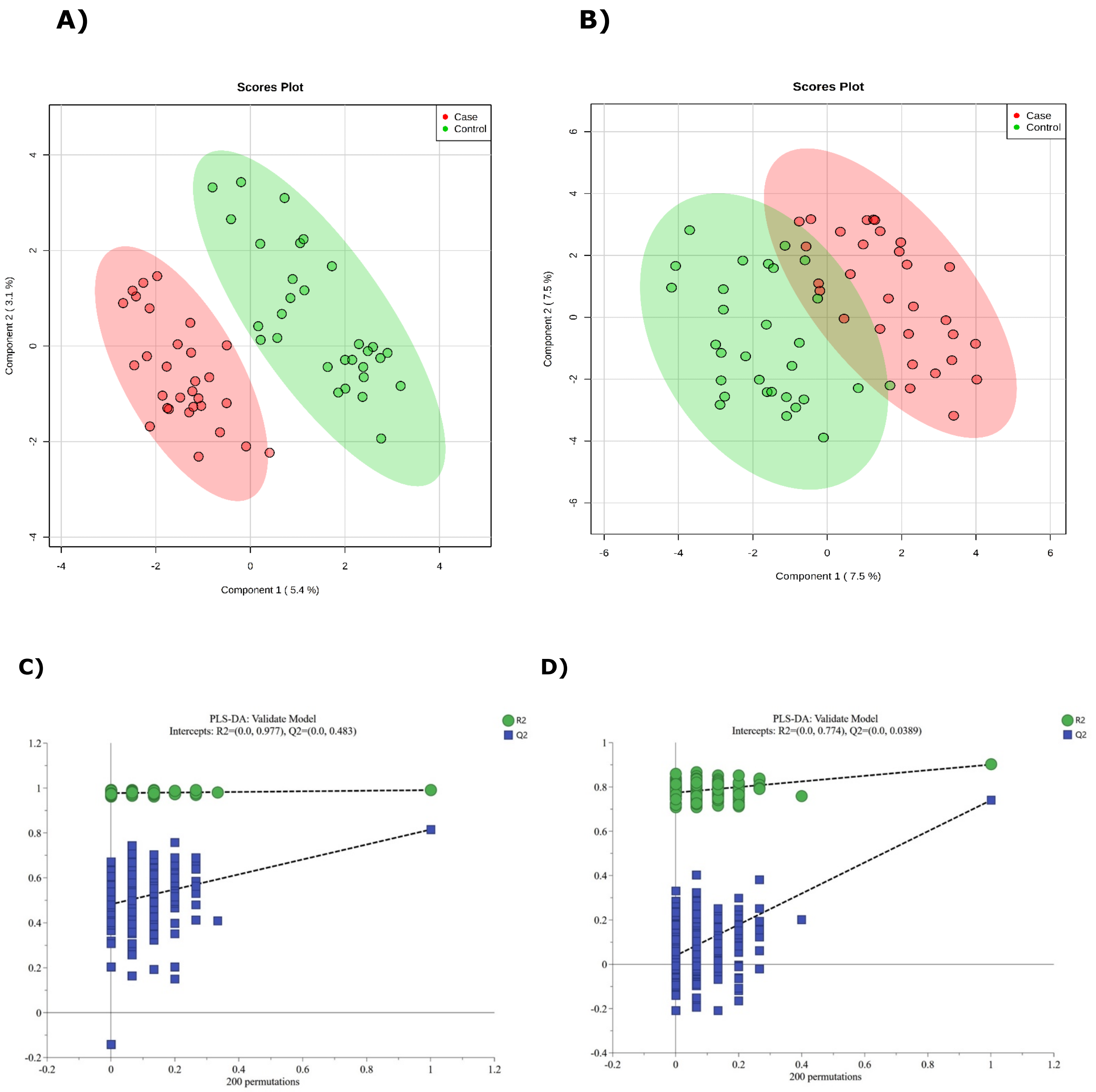

Supplementary Figure 5. The partial least squares-discriminant analysis (PLS-DA) model of metabolite profiling data. A**)** scores scatter plot of ESI negative mode, PC1 and PC2 can explain 5.4% and 3.1%, respectively, of the dataset total variance; B**)** scores scatter plot of ESI positive mode, PC1 and PC2 can explain 7.5% and 7.5%, respectively, of the dataset total variance. **C)** Permutation test in negative ion data. D**)** Permutation test in positive ion data.

A)

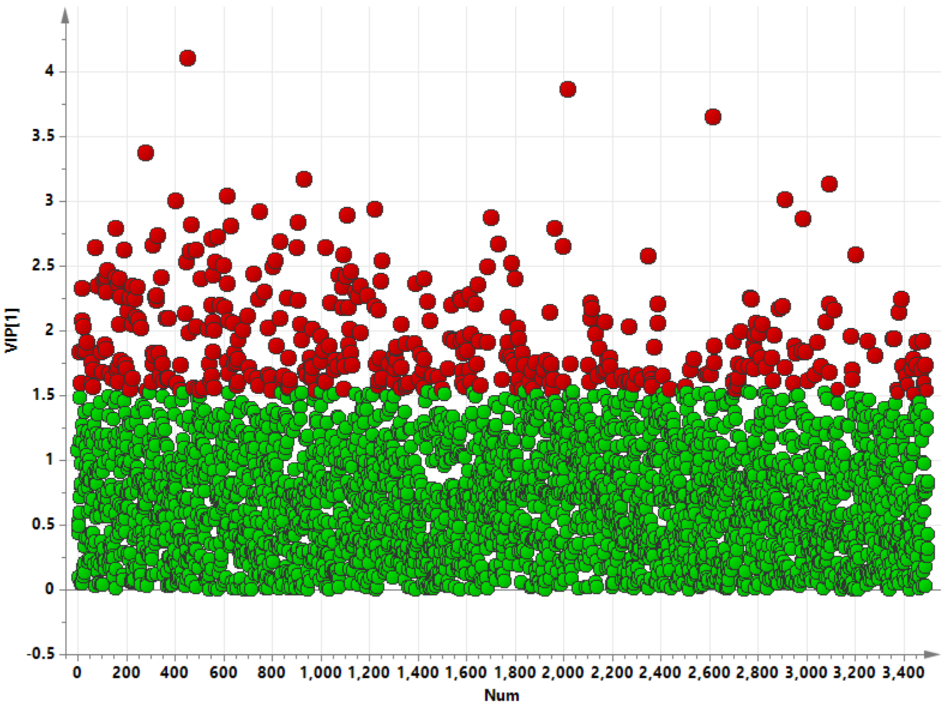

B)

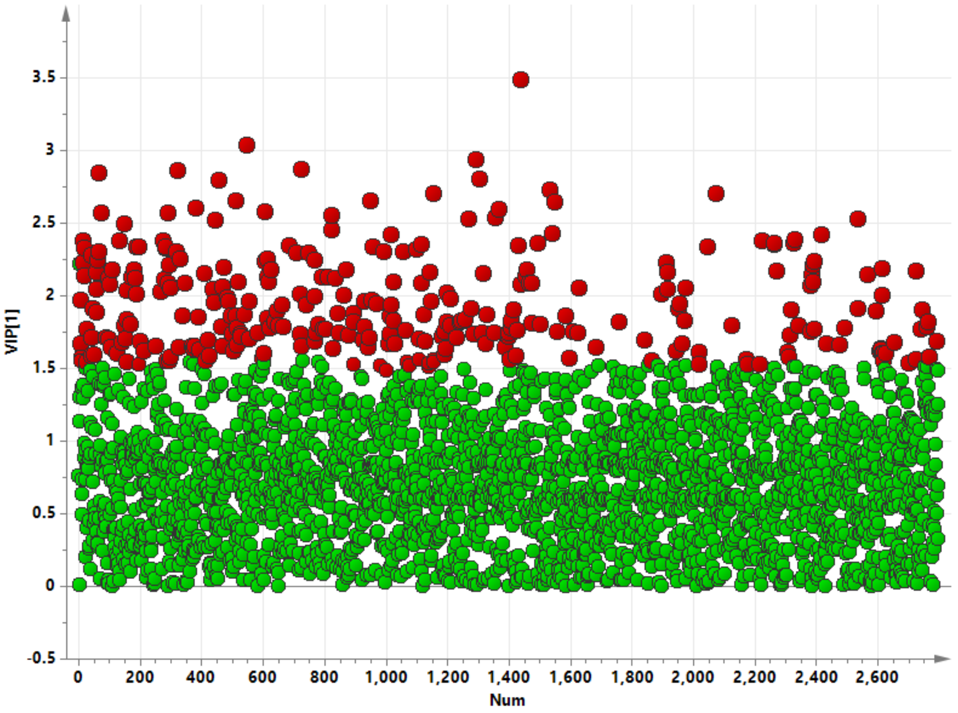

Supplementary Figure 6. The distribution of variable importance in projection (VIP) values (VIP>1.5). A) Electrospray ionization in negative mode; B) Electrospray ionization in positive mode.

A)

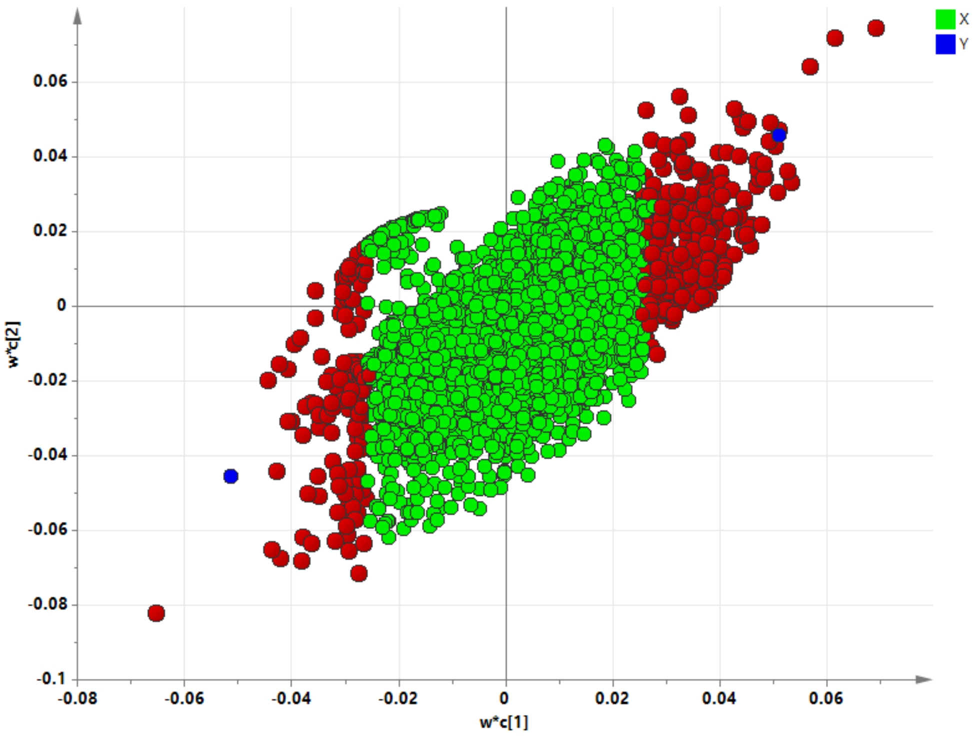

B)

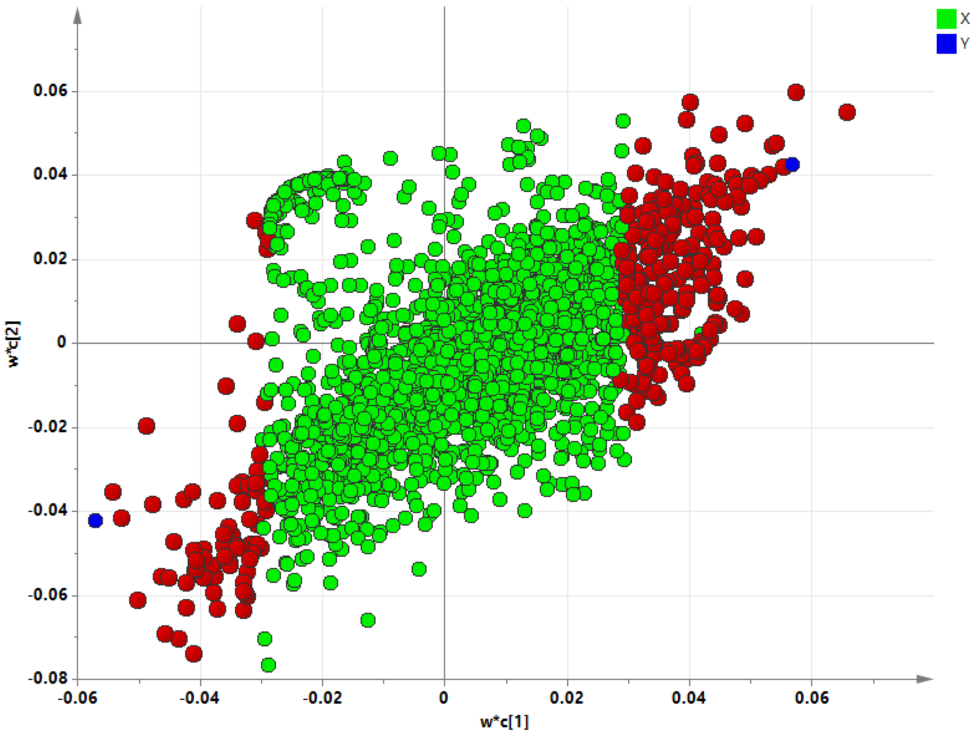

Supplementary Figure 7. The loading plot of PLS-DA model. The metabolites with red box were labeled as significant compounds (VIP＞1.5). A) ESI-mode; B) ESI+mode.

A)

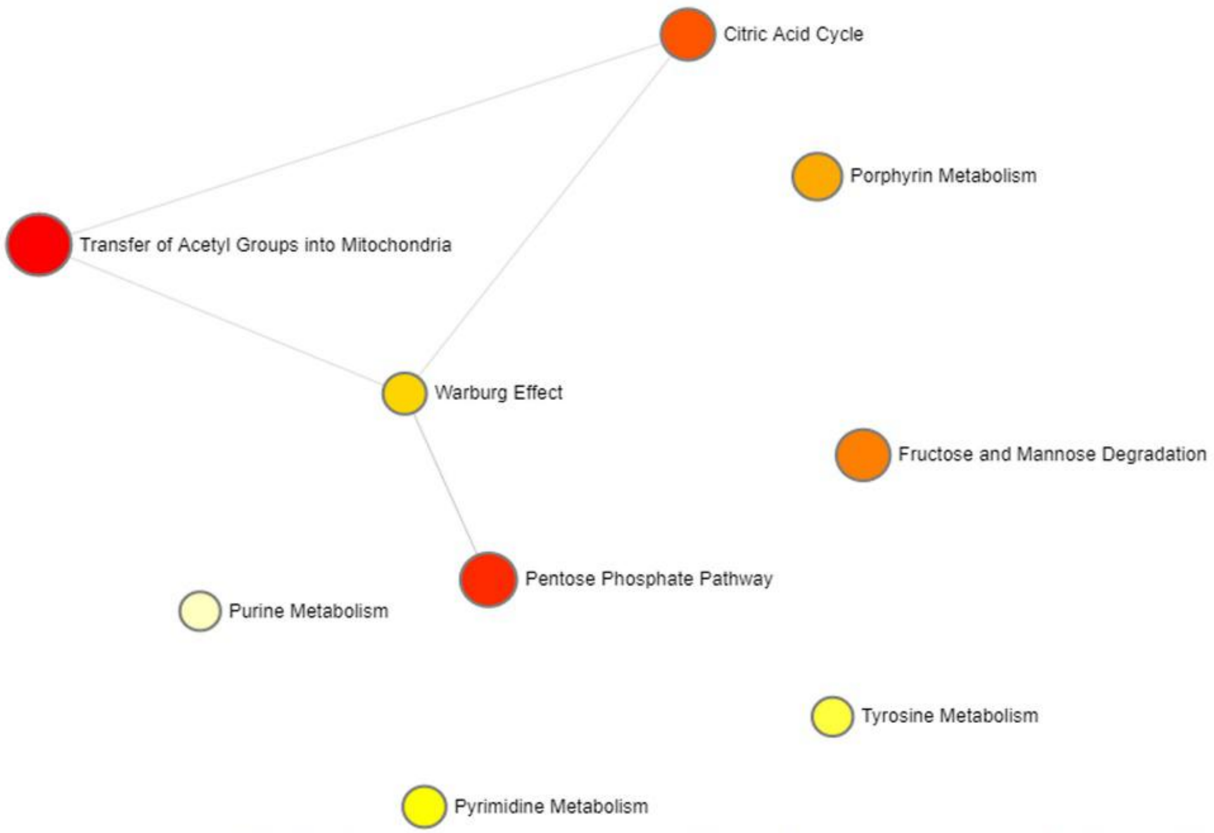

	Metabolite Set	Total	Hits	Expect	P value	Holm P	FDR
	Transfer of Acetyl Groups into Mitochondria	22	1	0.172	0.16	1.0	1.0
	Pentose Phosphate Pathway	29	1	0.227	0.206	1.0	1.0
	Citric Acid Cycle	32	1	0.25	0.225	1.0	1.0
	Fructose and Mannose Degradation	32	1	0.25	0.225	1.0	1.0
	Porphyrin Metabolism	40	1	0.312	0.274	1.0	1.0
	Warburg Effect	58	1	0.453	0.374	1.0	1.0
	Pyrimidine Metabolism	59	1	0.461	0.379	1.0	1.0
	Tyrosine Metabolism	72	1	0.562	0.443	1.0	1.0
	Purine Metabolism	74	1	0.578	0.452	1.0	1.0

B)

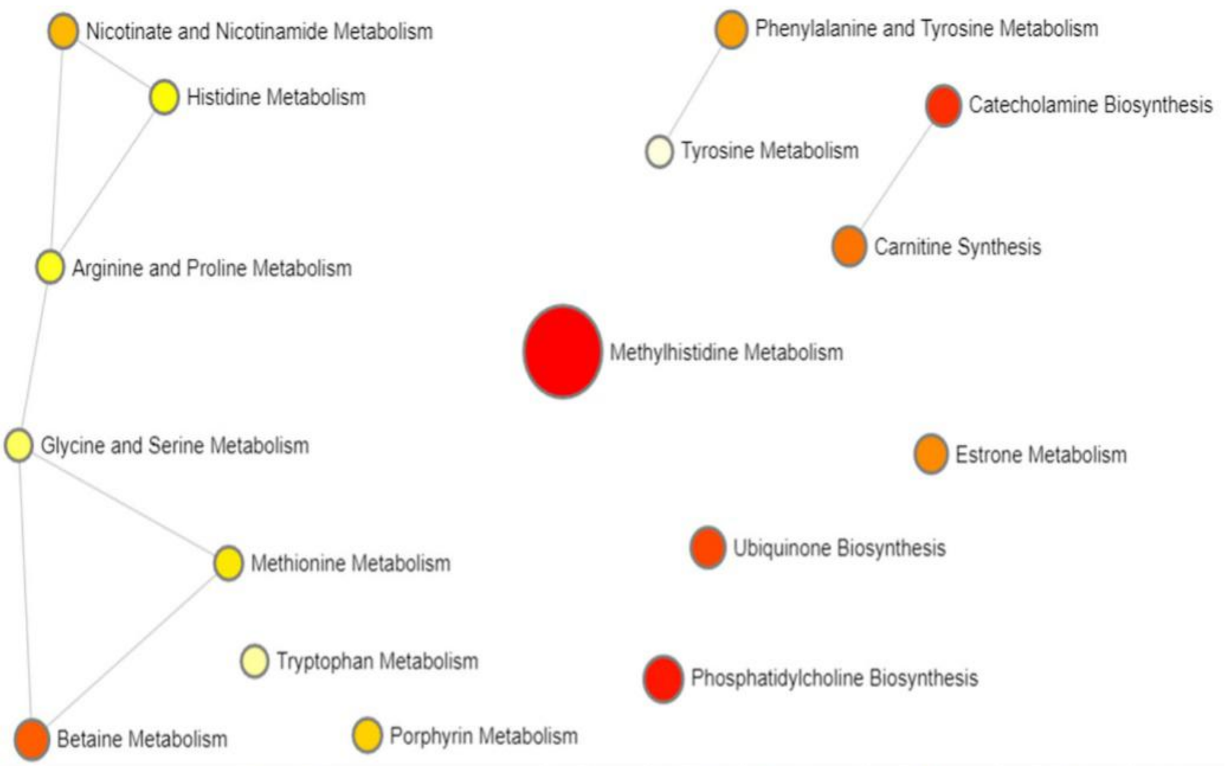

	Metabolite Set	Total	Hits	Expect	P value	Holm P	FDR
	Methylhistidine Metabolism	4	1	0.0469	0.0461	1.0	1.0
	Phosphatidylcholine Biosynthesis	14	1	0.164	0.153	1.0	1.0
	Catecholamine Biosynthesis	20	1	0.234	0.212	1.0	1.0
	Ubiquinone Biosynthesis	20	1	0.234	0.212	1.0	1.0
	Betaine Metabolism	21	1	0.246	0.221	1.0	1.0
	Carnitine Synthesis	22	1	0.258	0.231	1.0	1.0
	Estrone Metabolism	24	1	0.281	0.249	1.0	1.0
	Phenylalanine and Tyrosine Metabolism	28	1	0.328	0.284	1.0	1.0
	Nicotinate and Nicotinamide Metabolism	37	1	0.434	0.359	1.0	1.0
	Porphyrin Metabolism	40	1	0.469	0.382	1.0	1.0
	Methionine Metabolism	43	1	0.504	0.404	1.0	1.0
	Histidine Metabolism	43	1	0.504	0.404	1.0	1.0
	Arginine and Proline Metabolism	53	1	0.621	0.473	1.0	1.0
	Glycine and Serine Metabolism	59	1	0.691	0.511	1.0	1.0
	Tryptophan Metabolism	60	1	0.703	0.517	1.0	1.0
	Tyrosine Metabolism	72	1	0.844	0.585	1.0	1.0

Supplementary Figure 8. Pathway enrichment analysis of perturbed metabolites. A) At negative ion mode B) At positive ion mode.
